# Wound drains in posterior spinal surgery: a meta-analysis

**DOI:** 10.1186/s13018-016-0351-8

**Published:** 2016-01-22

**Authors:** Yancheng Liu, Yaomin Li, Jun Miao

**Affiliations:** Department of Spine Surgery, Tianjin Hospital, Tianjin, 300211 People’s Republic of China; Department of Rehabilitation, Tianjin Hospital, Tianjin, 300211 People’s Republic of China

**Keywords:** Spine, Posterior, Drain, Meta-analysis

## Abstract

**Background:**

The use of drains following posterior spinal surgery is controversial. Thus, the aim of this meta-analysis was to review the advantages and adverse effects of closed suction drainage systems in posterior spinal surgery.

**Methods:**

All randomized and non-randomized controlled trials comparing the use of closed suction drainage with no drainage in posterior spinal surgery were sought in PubMed, Medicine, Embase, and other Internet databases. All of the literature was searched and assessed by two independent reviewers, according to the standards of Cochrane systematic reviews. Data on functional and radiological outcomes in the two groups were pooled, which were then analyzed with RevMan software, version 5.2.

**Results:**

Four randomized controlled trials (RCTs) and four non-RCTs met the inclusion criteria. Meta-analysis revealed that no significant differences were found regarding wound infection (*P* = 0.83), hematoma (*P* = 0.48), neurological injury (*P* = 0.21), estimated blood loss (*P* = 0.59), or dry and moderate dressing drainage between the groups. The number of patients with saturated dressings was larger in the no drainage group (*P* = 0.002).

**Conclusions:**

There is no obvious evidence to support the application of closed suction drains for posterior spinal surgery. Because of the limited quality of the evidence currently available, more high-quality RCTs with better experimental designs and larger patient samples should be performed.

## Background

### Level of evidence: III

Closed suction drainage is commonly used in orthopedic surgery. The aim of using closed suction drainage is the prevention of the formation of hematomas [1]. Postoperative hematoma in the operative field can increase tension on incisions, delay wound healing, and lead to wound infection [2]. Moreover, epidural hematoma can lead to spinal cord compression and even paralysis in spinal surgery [3–5]. However, a few studies have demonstrated that closed suction drainage has no benefit in joint arthroplasty and spinal surgery [6, 7]. In contrast, closed suction drainage could cause retrograde infection, increase postoperative blood loss, and the need for transfusion [8, 9].

The use of closed suction drainage in posterior spinal surgery remains controversial [10–15]. Therefore, we conducted a meta-analysis, pooling the data from randomized controlled trials (RCTs) and non-RCTs to provide an evidence-based judgment regarding the use of closed suction drainage in posterior spinal surgery.

## Methods

### Search strategy

**Fig. 1:**
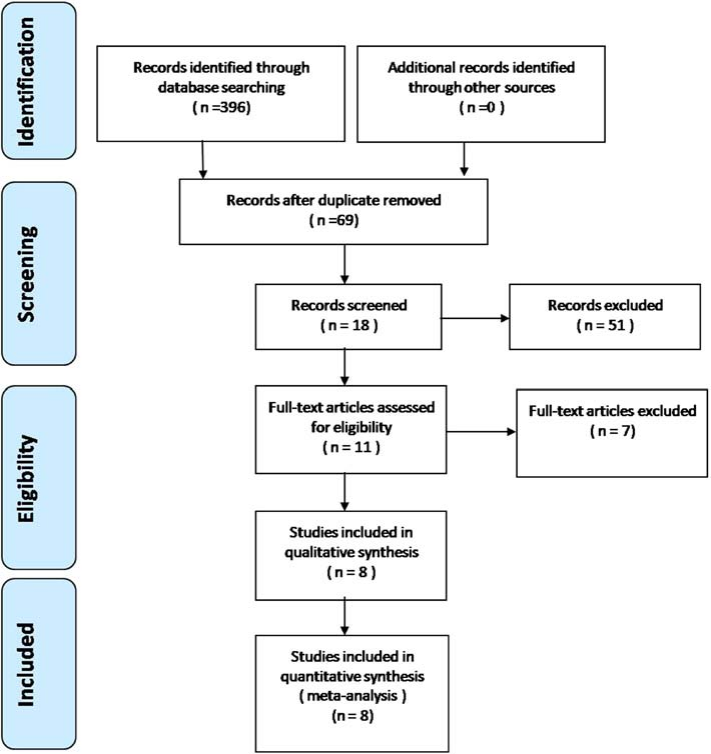
Flowchart of the study selection process

Electronic databases, including the Cochrane Library, Medline (1966–2015.10), PubMed (1966–2015.10), Embase (1980–2015.10), and ScienceDirect (1985–2015.10), were searched. Gray studies were identified from the references of the included literature. No language restrictions were applied. The search process was conducted as illustrated in Fig. 1. The keywords “Drain OR Drainage”, “spine OR spinal,” and “posterior” were used in combination with the Boolean operators AND and OR.

### Selection criteria

Studies were considered eligible for inclusion if they met the following criteria: (1) the patients underwent primary posterior spinal surgery, (2) the intervention was the use of drainage compared to no drainage, (3) the outcomes included blood loss, transfusion, and complication, and (4) the study was a published or unpublished controlled clinical trial.

Exclusion criteria: Patients were excluded from the meta-analysis if they had a neoplastic etiology (i.e., metastasis or myeloma), infection, traumatic fracture, serious osteoporosis, metal sensitivity, or mental illness.

### Quality assessment

Two reviewers completed the search process independently. Disagreement was resolved by consulting a third reviewer. Quality assessment for randomized trials was conducted according to a modification of the generic evaluation tool used by the Cochrane Bone, Joint and Muscle Trauma Group [16] and the index for non-randomized studies (MINORS) form for non-randomized clinical trials [17]. The methodological quality of each trial was scored from 0 to 24.

### Data extraction

Two researchers independently extracted the data from the included literature. In cases of incomplete data, the study authors were consulted for details. The following data were extracted: first author name, year of publication, intervening measures, comparable baseline data, sample size, and outcome measurements. Other relevant parameters were also extracted from individual studies.

### Data analysis and statistical methods

The pooled data were analyzed using RevMan software, version 5.1 (The Cochrane Collaboration, Oxford, United Kingdom). Heterogeneity was estimated depending on the values of *P* and *I*^2^ using the standard chi-square test. When *I*^2^ > 50 %, *P* < 0.1 was considered to indicate significant heterogeneity. Therefore, a random-effects model was applied for data analysis. A fixed-effects model was used when no significant heterogeneity was found. In cases of significant heterogeneity, subgroup analysis was performed to investigate sources. For continuous outcomes, mean differences (MDs) and 95 % confidence intervals (CIs) are presented. Risk difference (RD) and 95 % CIs were calculated for dichotomous data.

## Results

### Literature search

A total of 396 potential studies were identified with the primary search strategy. Of these studies, 388 reports were excluded according to the eligibility criteria. No additional studies were obtained after the reference review. Ultimately, four non-RCTs and four RCTs [10–15, 18, 19] were eligible for data extraction and meta-analysis, as indicated by the flowchart in Fig. 1. These studies involved a total of 1133 patients in the drainage group and 771 patients in the no drainage group.

### Study characteristics

The main characteristics of the included studies are reported in Table 1. Statistically, similar baseline characteristics were observed between the two groups. The sample sizes of included studies ranged from 30 to 560 patients. The surgical procedures of the four studies were single-level lumbar decompression surgeries [13–15, 18]. The surgical procedures of three studies were posterior spinal fusion and instrumentation [10, 12, 19]. In Brown’s studies, the surgical procedure was extensive lumbar spine surgery [11].

**Table 1:** Characteristics of included studies

Studies	Design	Cases (D/C)	Mean age (D/C)	Male (D/C)	Surgical procedure/patient population	Follow-up
Payne et al. [14]	RCT	103/97	NA	NA	Single-level lumbar laminectomy	2 weeks
Blank et al. [10]	RCT	18/12	13.9	18	Posterior spinal fusion and instrumentation	Discharge
Brown and Brookfield [11]	RCT	42/41	67.4/67.4	NA	Extensive lumbar spine surgery	Discharge
Sen et al. [15]	PCT	41/38	46.4	45	Unilateral, single-level lumbar disc herniation	6–12 months
Mirzai et al. [18]	RCT	22/28	47/47	16/17	Hemipartial laminectomy and flavectomy	6 months
Kanayama et al. [13]	CCT	298/262	44/48	190/168	Single-level lumbar decompression surgery	Discharge
Walid 2011	CCT	285/117	57.3	173	Posterior lumbar interbody fusion	Discharge
Diab et al. [12]	PCT	324/176	15.7/15.6	59/43	Posterior spinal fusion and instrumentation	2 years

*D* drainage, *C* no drainage, *RCT* randomized controlled trial, *PCT* prospective controlled trial, *CCT* case controlled trial, *NA* no available

### Risk of bias assessment

**Fig. 2:**
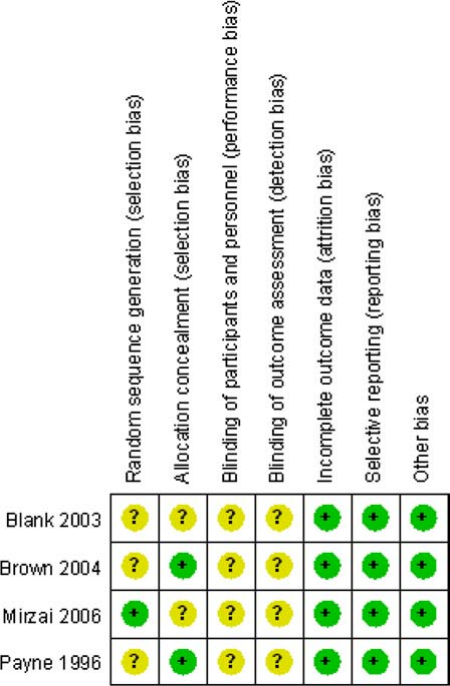
The summary of bias risk of randomized controlled trials

The quality of the RCTs was assessed according to the Cochrane Handbook for Systematic Review of Interventions. Four RCTs met the inclusion criteria. Mirzai et al. reported that randomization was performed by flipping a coin; the three other RCTs provided randomization methods. Adequate concealment of allocation was unclear for two RCTs [10, 18]. None of the RCTs reported blinding methods. The MINORS scores were 17–19 for the non-RCTs [12, 13, 15, 19]. The methodological quality assessment is illustrated in Fig. 2 (RCTs) and Table 2 (non-RCTs).

**Table 2:** Quality assessment for non-randomized trials

Quality assessment for non-randomized trials	Sen et al. [15]	Kanayama et al. [13]	Walid 2011	Diab et al. [12]
A clearly stated aim	2	2	2	2
Inclusion of consecutive patients	2	1	1	2
Prospective data collection	2	2	2	2
Endpoints appropriate to the aim of the study	1	1	1	1
Unbiased assessment of the study endpoint	1	1	1	0
A follow-up period appropriate to the aims of study	2	2	2	2
Less than 5 % loss to follow-up	2	2	2	2
Prospective calculation of the sample size	0	0	0	0
An adequate control group	2	2	2	2
Contemporary groups	1	0	1	1
Baseline equivalence of groups	2	2	2	2
Adequate statistical analyses	2	2	2	1
Total score	19	17	18	17

### Outcomes for meta-analysis

#### Wound infection

**Fig. 3:**
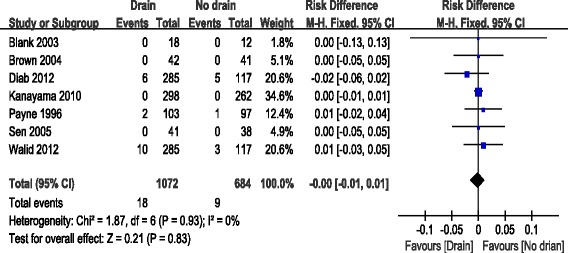
Forest plot of wound infection between the two groups

Details regarding wound infection were available in seven studies [10–15, 19]. There was significant heterogeneity (*χ*^2^ = 1.87, *df* = 6, *I*^2^ = 0 %, *P* = 0.93); therefore, a fixed model was applied. Pooling of the results demonstrated that wound infection showed no significant difference between the two groups (RD = −0.0; 95 % CI, −0.01 to 0.01; *P* = 0.83; Fig. 3).

#### Hematoma

**Fig. 4:**
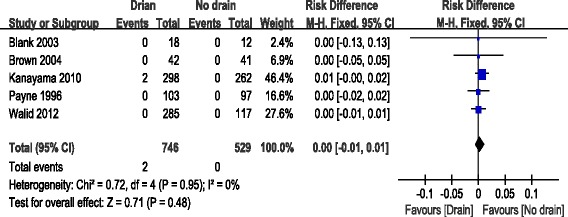
Forest plot of hematoma between the two groups

Five articles reported the incidence of hematoma [10, 11, 13, 14, 19]. Significant heterogeneity was found, so a fixed model was used (*χ*^2^ = 0.72, *df* = 4, *I*^2^ = 0 %, *P* = 0.72). There was no significant difference between the drain group and the no drain group regarding hematoma (RD = 0.0; 95 % CI, −0.01 to 0.01; *P* = 0.48; Fig. 4).

#### Postoperative neurological injury

**Fig. 5:**
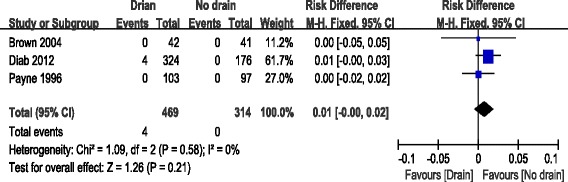
Forest plot of postoperative neurological injury between the two groups

Relevant data regarding postoperative neurological injury were documented in three articles [11, 12, 14]. Significant heterogeneity was found, so a fixed model was used (*χ*^2^ = 1.09, *df* = 2, *I*^2^ = 0 %, *P* = 0.58). There was no significant difference between the drain group and the no drain group regarding postoperative neurological injury (RD = 0.01; 95 % CI, −0.00 to 0.02, *P* = 0.21; Fig. 5).

#### Estimated blood loss

**Fig. 6:**
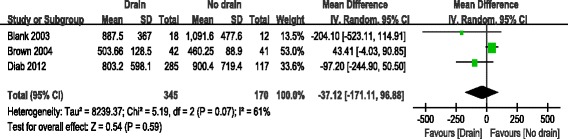
Forest plot of estimated blood loss between the two groups

Three studies reported estimated blood loss [10–12]. Significant heterogeneity was found, so a random-effects model was used (*χ*^2^ = 5.19, *df* = 2, *I*^2^ = 61 %, *P* = 0.07). There was no significant difference between the drain group and the no drain group regarding postoperative neurological injury (MD = −37.12; 95 % CI, −171.11 to 96.88; *P* = 0.59; Fig. 6).

#### Postoperative dressing saturation

Postoperative dressing saturation was reported in two trials [10, 11]. For dry dressing drainage, significant heterogeneity was shown between the pooled results; thus, a random-effects model was performed. There was no significant difference between the groups (RD = 0.19; 95 % CI, −0.20 to 0.58; *P* = 0.33). For moderate dressing drainage, significant heterogeneity was shown between the pooled results; thus, a random-effects model was performed. There was no significant difference between the groups (RD = 0.00; 95 % CI, −0.40 to 0.40; *P* = 0.99). For saturated dressing drainage, no significant heterogeneity was shown between the pooled results; thus, a fixed model was performed. There were significant differences between the groups (RD = −0.23; 95 % CI, −0.38 to −0.09; *P* = 0.002).

## Discussion

The most important finding of the present meta-analysis was that the use of drainage in posterior spinal surgery decreased saturated dressing drainage, but it did not reduce postoperative wound infection, hematoma, neurological injury, or estimated blood loss. Furthermore, no significant differences were found in dry or moderate dressing drainage.

Followed strict searching, four non-RCTs [12, 13, 15, 19] and four RCTs [10, 11, 14, 18] met the inclusion criteria for the meta-analysis. There were biases for randomization, concealment of allocation, and blinding methods in the RCTs. The quality assessment scores of non-RCTs ranged from 17 to 19. No prospective calculation of the sample sizes was described in the non-RCTs. In addition, the contemporary groups were biased. All of these shortcomings weakened the level of evidence and should be considered when interpreting the findings of the present meta-analysis.

Wound infection is a common complication after posterior spinal surgery, increasing morbidity and medical costs. Spinal surgeons have advocated closed suction drainage due to a fear of infection [20, 21]. The present meta-analysis found no significant difference in the incidence of wound infection. The incidence of infection was 1.68 % in the closed suction drainage group and 1.32 % in the no drainage group.

In theory, a hematoma in the wound is an excellent culture medium for bacterial growth. In spinal surgery, symptomatic epidural hematomas can cause spinal cord compression and even paralysis [22]. The present meta-analysis found no significant difference in the incidence of hematomas. In Mirzai et al.’s study, epidural hematomas were measured by magnetic resonance imaging (MRI) examinations [18]. They found that the group with drains had significantly fewer patients with hematomas and significantly more patients with no hematomas. However, none of the hematomas had significant effects on the recovery of any patients.

Two of the included studies reported postoperative dressing saturation from the wounds [10, 11]. The pooled data demonstrated that the number of patient with saturated dressings was larger in the no drainage group. Saturated dressings reflected leakage of blood from wounds without drainage without the formation of hematoma.

Some studies showed that drainage was associated with significant blood loss and transfusion requirements [19]. In our meta-analysis, the pooled data demonstrated that drainage did not increase blood loss. Blank et al. reported that the transfusion requirements were similar for both groups [10]. In Walid et al.’s study, an increased rate of allogeneic blood transfusion was noted with posthemorrhagic anemia and drain use [19].

There were several potential limitations of our meta-analysis: (1) only four RCTs and four non-RCTs were identified, and the sample sizes of the included studies were relatively small; (2) there were some methodological weaknesses in the included studies; and (3) some data were incomplete, and we failed to conduct meta-analysis of factors such as transfusion requirements.

## Conclusions

In summary, the use of drainage in posterior spinal surgery did not decrease infection, hematoma, or postoperative neurological injury. There was no obvious evidence to support the application of closed suction drains for posterior spinal surgery. Because of the limited quality of the evidence currently available, more high-quality RCTs with better experimental designs and larger patient samples should be performed.
